# Molecular and Clinical Analyses of 111 Patients with Bilateral Anterior-Segment Dysgenesis/Aniridia and Microphthalmia/Anophthalmia

**DOI:** 10.1016/j.xops.2026.101339

**Published:** 2026-07-28

**Authors:** Sachiko Nishina, Hazuki Anzai, Tomoyo Yoshida, Yoshito Koyanagi, Sayaka Kamada, Kumiko Kato, Kaoruko Torii, Akiko Hikoya, Mineo Kondo, Chie Sotozono, Keiko Matsubara, Maki Fukami

**Affiliations:** 1 Division of Ophthalmology, National Center for Child Health and Development, Tokyo, Japan; 2 Center for Medical Genetics, National Center for Child Health and Development, Tokyo, Japan; 3 Department of Ophthalmology, Kyoto Prefectural University of Medicine, Kyoto, Japan; 4 Department of Ophthalmology, Mie University Graduate School of Medicine, Tsu, Japan; 5 Department of Ophthalmology, Hamamatsu University School of Medicine, Hamamatsu, Japan; 6 Division of Diversity Research, National Research Institute for Child Health and Development, Tokyo, Japan; 7 Department of Molecular Endocrinology, National Research Institute for Child Health and Development, Tokyo, Japan

**Keywords:** Anterior segment dysgenesis, Aniridia, Microphthalmia, Anophthalmia, Panel sequencing

## Abstract

**Objective:**

To clarify the molecular and clinical characteristics of anterior-segment dysgenesis (ASD)/aniridia and microphthalmia/anophthalmia caused by monogenic variants.

**Design:**

Clinical and genetic analyses of a large cohort of patients with bilateral ocular lesions.

**Participants:**

A total of 111 patients and their family members were recruited through a multicenter collaborative study in Japan.

**Methods:**

Next-generation sequencing using custom-designed panels for 11 and 12 major causative genes for ASD/aniridia and microphthalmia/anophthalmia, respectively. We analyzed the clinical information of patients with pathogenic or likely pathogenic variants.

**Main Outcome Measures:**

Collated genetic results and clinical data.

**Results:**

We achieved genetic diagnosis rates of 50.0% for ASD/aniridia and 37.5% for microphthalmia/anophthalmia. We identified 11 previously unreported variants. De novo variants in the *PAX6*, *PITX2*, or *GJA8* genes and parentally derived variants in the *FOXC1* and *CYP1B1* genes were the major causes of ASD/aniridia. Microphthalmia/anophthalmia‑associated variants in the *ABCB6*, *BMP4*, and *OTX2* genes were predominantly inherited from parents with no or different ocular phenotypes. We observed phenotypic diversity and variable ocular and systemic complications in variant-positive patients. Most importantly, this study showed a high incidence of glaucoma in patients with *CYP1B1* and *FOXC1* variants and frequent systemic abnormalities in patients with *FOXC1* and *PITX2* variants. In addition, *BMP4* and *RARB* variants were associated with neurologic abnormalities.

**Conclusions:**

This study provides evidence that targeted gene panel approaches are useful for the clinical diagnosis of ASD/aniridia and microphthalmia/anophthalmia. Our data clarified the mutation spectrum and phenotypic characteristics of these disorders caused by monogenic variants. This study confirmed that various phenotypes classified as ASD, such as Peters anomaly and Axenfeld anomaly, constitute a group of disorders on the same spectrum, may overlap with aniridia, and exhibit genetic heterogeneity. The findings, which demonstrate an association between genotypes and complications, are expected to contribute to better management and care for children with these rare intractable eye diseases.

**Financial Disclosure(s):**

Proprietary or commercial disclosure may be found in the Footnotes and Disclosures at the end of this article.

Anterior-segment dysgenesis (ASD), aniridia, and microphthalmia/anophthalmia are intractable eye diseases that cause severe visual impairment from infancy. Anterior-segment dysgenesis is a term that recently has come into use to describe congenital anomalies of the ocular anterior segment as a spectrum of disorders that are classified into distinct stages, including posterior embryotoxon, Axenfeld anomaly, posterior keratoconus, Peters anomaly, sclerocornea, and anterior staphyloma. Aniridia is a congenital disorder characterized by complete or partial absence of the iris; although it is considered a separate condition from ASD, it is similarly associated with various complications.

To register ASD and aniridia as designated intractable diseases, clinical studies conducted throughout Japan, and diagnostic criteria and severity grading have been reported in accordance with established standards.^1^, ^2^, ^3^, ^4^, ^5^ Microphthalmia is similarly classified as a rare disease of infancy and childhood and is a noteworthy condition for which results from a nationwide survey in Japan have been reported.^6^ Monogenic mutations play a major role in the development of these disorders. More than 50 and 80 genes have been implicated in the development of ASD/aniridia and microphthalmia/anophthalmia, respectively.^7^ Accurate genetic diagnosis contributes to better management of ocular and systemic complications, low-vision care, and genetic counseling for patients with these diseases. However, molecular tests for these diseases in infants and young children are not generally applied in clinical practice. Hence, the frequencies of each monogenic mutation and genotype-phenotype correlations remain largely unknown.

We performed molecular and clinical analyses for 111 patients (102 families) who had bilateral ASD/aniridia or microphthalmia/anophthalmia. This study is the first to investigate the genetic background of ASD/aniridia and microphthalmia/anophthalmia in East Asian populations. These patients were recruited through the collaboration of 4 major institutes specializing in pediatric ophthalmology in Japan. We used custom next-generation sequencing (NGS) panels for the molecular diagnosis of patients. We also investigated genotype-phenotype correlations and associated complications in patients with monogenic abnormalities.

## Methods

### Study Design

We formed a multicenter collaborative research group comprised of 4 institutions: the National Center for Child Health and Development, Kyoto Prefectural University of Medicine, Mie University Graduate School of Medicine, and Hamamatsu University School of Medicine. Patients diagnosed with ASD/aniridia and microphthalmia/anophthalmia were recruited in these facilities between August 2024 and October 2025. The Central Institutional Review Board of the National Center for Child Health and Development approved this study (No. 2024-007), which adhered to the tenets of the Declaration of Helsinki. The other institutions provided research implementation permits. The patients and/or their parents provided written informed consent to participate in this study. Genetic counseling was provided to patients and their parents before genetic test and upon reporting of the results.

### Participants

The initial inclusion criteria were patients clinically diagnosed with bilateral ASD, aniridia, microphthalmia, or anophthalmia. Cases with unilateral lesions were excluded, but cases with differing clinical diagnoses between the left and right eyes and cases with varying degrees of severity between the left and right eyes were included. The study primarily targeted Japanese individuals but also included a small number of patients of East Asian ethnicity residing in Japan. Individuals with pathogenic chromosomal abnormalities were excluded.

The study group was comprised of 97 patients (88 families) with ASD and/or aniridia, and 14 patients (14 families) with microphthalmia/anophthalmia, including a case of microphthalmia with a *RARB* variant reported in our recent study.^8^ Clinical diagnoses were determined according to the Japanese diagnostic criteria.^1^^,^^2^^,^^6^ The ages of the patients at the time of genetic testing ranged from 1 month to 69 years. Pediatric patients aged ≤18 years accounted for 84.7% (94 cases), and those aged ≤6 years accounted for 39.6% (44 cases).

### Clinical Analyses

We investigated the clinical records and phenotypes of the anterior segments of 111 patients. We collected detailed information on ocular and systemic complications and the phenotypes of the patients’ parents.

### Genetic Diagnosis

Genomic DNA was extracted from the peripheral leukocytes of patients. Because this study aimed to establish an efficient NGS-based diagnosis method for ASD/aniridia and microphthalmia/anophthalmia, we focused on major known causative genes for these disorders. Based on previous reports,^9^, ^10^, ^11^, ^12^, ^13^, ^14^ we selected 11 genes (*B3GLCT*, *COL4A1*, *CPAMD8*, *CYP1B1*, *FOXC1*, *FOXE3*, *GJA8*, *PAX6*, *PITX2*, *PITX3*, and *PXDN*) for the NGS panel of ASD/aniridia, and 12 genes (*ABCB6*, *ALDH1A3*, *BMP4*, *CRYBA4*, *GDF3*, *OTX2*, *RARB*, *RAX*, *SOX2*, *STRA6*, *TENM3*, and *VSX2*) for the microphthalmia/anophthalmia panel. The modes of inheritance of the pathogenic variants in these genes are shown in Supplemental Table 1 (available at www.ophthalmologyscience.org). Short-read NGS for the coding regions of these genes was performed at the Kazusa DNA Research Institute (Kisarazu, Japan) as described previously.^15^ We searched for rare variants whose allele frequencies in gnomAD (https://gnomad.broadinstitute.org/) and ToMMo_8.3KJPN (https://www.megabank.tohoku.ac.jp/) were <0.01. The selected variants were annotated using the ClinVar (https://www.ncbi.nlm.nih.gov/clinvar) and Human Gene Mutation Database (https://www.hgmd.cf.ac.uk/ac/index.php). The functional outcomes of the variants were predicted by in-silico analyses using Mutation Taster (https://www.mutationtaster.org/), SIFT (https://sift.bii.a-star.edu.sg/), and PolyPhen2 (https://bio.tools/polyphen-2). When possible, we performed Sanger sequencing for the parents of variant-positive patients to examine the parental origin of the variants. We referred to the standards and guidelines for the interpretation of sequence variants of the American College of Medical Genetics and Genomics to assess the pathogenicity of each variant.^16^

## Results

### ASD/Aniridia

**Figure 1:**
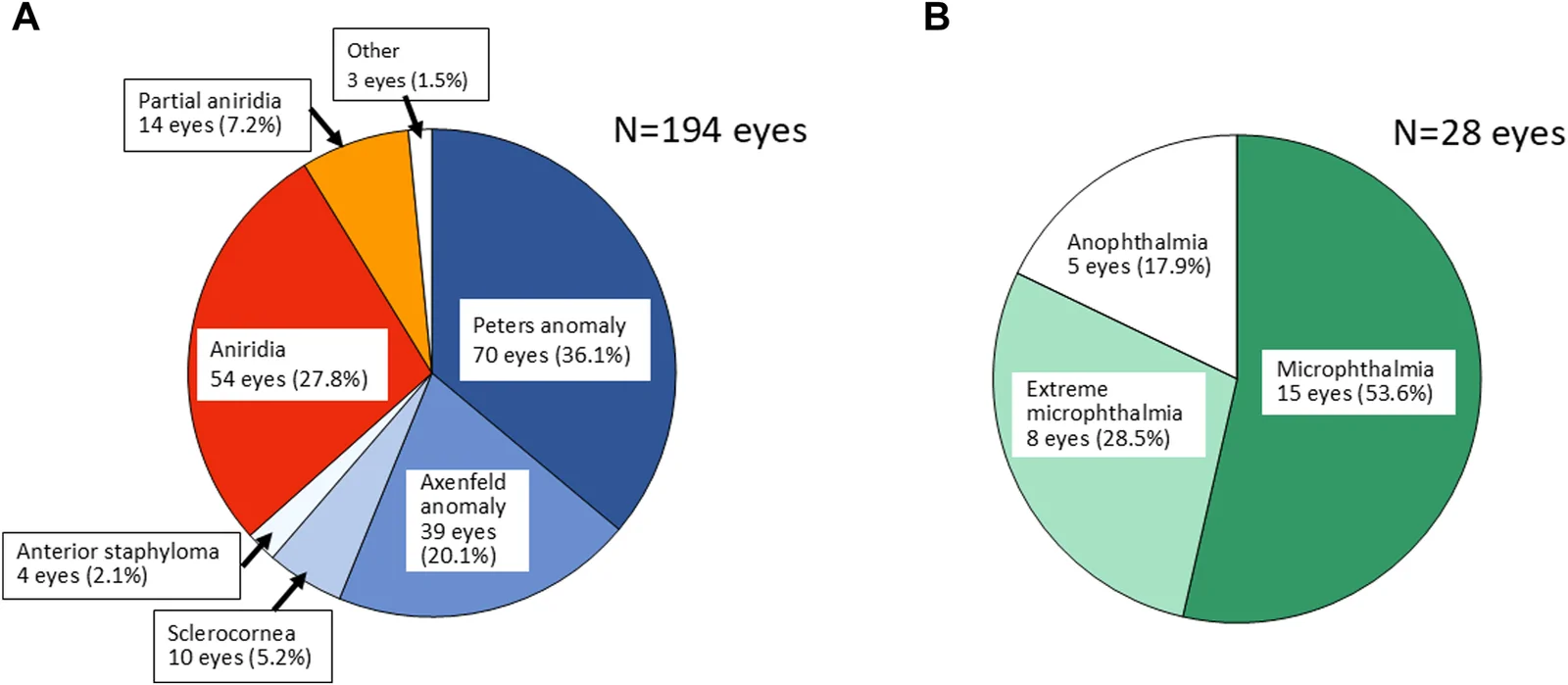
**A,** Clinical diagnoses of 194 eyes of 97 patients with anterior-segment dysgenesis/aniridia. Anterior-segment dysgenesis accounts for 63.4% and aniridia for 35.1%. The most common diagnosis was Peters anomaly followed by aniridia, Axenfeld anomaly, partial aniridia, sclerocornea, and anterior staphyloma. Others included 3 eyes each of extreme microphthalmos, congenital cataract, and glaucoma in the other eye of bilateral cases. **B,** The clinical diagnosis of 28 eyes of 14 index patients with microphthalmia. Based on severity, the affected eyes were classified as having microphthalmia, extreme microphthalmia, or anophthalmia.

The clinical diagnoses of 194 eyes of 97 index patients with ASD/aniridia are shown in Figure 1A. Of the 97 patients, 11 (11.3%) exhibited different phenotypes in the right and left eyes. Anterior-segment dysgenesis, including Peters anomaly, Axenfeld anomaly, sclerocornea, and anterior staphyloma, accounted for 123 eyes (63.4%), while aniridia and partial aniridia accounted for 68 eyes (35.1%). Ocular complications and/or associated ocular anomalies were observed in 121 eyes (62.4%), and systemic abnormalities were present in 29 patients (29.9%) (Supplemental Table 2, available at www.ophthalmologyscience.org). Twenty-seven patients (27.8%) had a family history, 8 of which had phenotypes that differed within the family.

We identified possible pathogenic variants in 6 genes in 44 of 88 families, yielding a molecular diagnostic rate of 50.0%. Table 1 shows the causative genes identified for clinical diagnoses. Multiple causative genes have been identified for clinical diagnoses. The genetic diagnosis rate was high at 54 of 68 eyes (79.4%) for aniridia and partial aniridia but low at 44 of 123 eyes (35.8%) for ASD.

**Table 1:** Pathogenic Variants Identified in Patients with ASD/Aniridia (194 Eyes of 97 Patients)

Clinical Diagnosis∗ (No. of Eyes)	Affected Genes (No. of Eyes)	No. of Eyes with Pathogenic Variants (Diagnostic Rate)
Peters anomaly (70)	*CYP1B1* (12) *PITX3* (4) *FOXC1* (2) *PAX6* (2)	20 (28.5%)
Axenfeld anomaly (39)	*PITX2* (12) *FOXC1* (6) *GJA8* (2), *CYP1B1* (2) *PAX6* (1)	23 (60.0%)
Sclerocornea (10)	(–)	0 (0.0%)
Anterior staphyloma (4)	*FOXC1* (1)	1 (25.0%)
Aniridia (54)	*PAX6* (36) *CYP1B1* (3) *FOXC1* (2)	41 (75.9%)
Partial aniridia (14)	*PAX6* (6) *CYP1B1* (4) *FOXC1* (3)	13 (92.9%)
Others† (3)	*CYP1B1* (1) *PAX6* (1)	

ASD = anterior-segment dysgenesis.

∗ 11 of 97 patients exhibited different phenotypes in the right and left eyes.

† Others included glaucoma, cataract, and microphthalmia in one eye.

Detected variants are shown in Supplemental Table 3 (available at www.ophthalmologyscience.org). All 36 variants, except for 2 *CYP1B1* variants, were assessed as pathogenic or likely-pathogenic according to the American College of Medical Genetics and Genomics guidelines. The remaining *CYP1B1* variants were assessed as variants of unknown significance; however, all in-silico programs predicted the deleterious effects of these variants on the protein structure.

De novo occurrence was confirmed in 6 of 11 (54.5%) patients with *PAX6* variants and 5 of 7 (71.4%) patients with *FOXC1* variants. In the remaining cases, *PAX6* variants were inherited from a parent with the same phenotype, while the remaining 2 *FOXC1* variants were inherited from an unaffected mother. One of 2 variants in *PITX2* and 1 variant in *GJA8* were de novo, while 1 variant in *PITX3* was of paternal origin. Of the 22 *CYP1B1* variants in 11 patients, 21 were confirmed to have been derived from unaffected parents; paternal genetic data were unavailable for the remaining 1 patient. Of note, we identified several previously unreported variants, that is, 2 *PAX6* variants (c.141G>C and c.853_854del), 3 *FOXC1* variants (c.140_141dupAC, c.673del, and c.484T>C), and 3 *PITX2* variants (c.282del, c.232C>T, and c.138dup). Two *CYP1B1* variants (c.575A>T and c.512T>C) have been reported only in East Asia.

**Figure 2:**
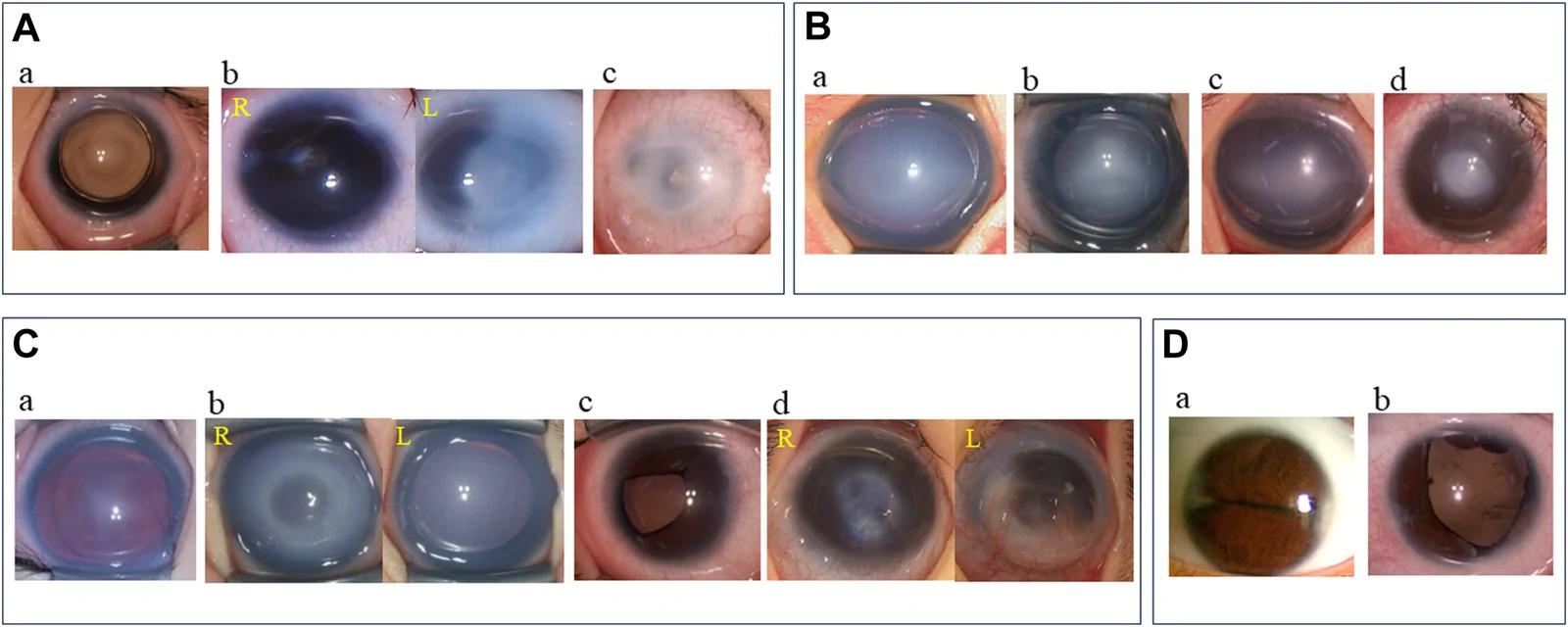
Photographs of the anterior segment associated with the causative gene (A indicates the patient identification number in Table 2). **A,***PAX6*-related phenotypes. **a.** Aniridia with glaucoma (L, A-1). **b.** Axenfeld anomaly with pupillary closure (R) and Peters anomaly (L) (A-13). **c.** Peters anomaly (R, A-10). **B,***CYP1B1*-related phenotypes. **a.** Aniridia with glaucoma (R, A-31). **b.** Peters anomaly with aniridia and glaucoma (R, A-32). **c.** Peters anomaly with partial aniridia and glaucoma (R, A-25). **d.** Peters anomaly with glaucoma (L, A-24). **C,***FOXC1*-related phenotypes. **a.** Aniridia with glaucoma (L, A-38). **b.** Peters anomaly with glaucoma (R) and aniridia with glaucoma (L) (A-39). **c.** Axenfeld anomaly with glaucoma (R) (A-37). **d.** Peters anomaly (R) and anterior staphyloma (L) (A-35). **D,***PITX2*-related phenotypes. **a.** Axenfeld anomaly (L) (A-43). **b.** Axenfeld anomaly (L) (A-44). L = left; R = right.

Table 2 shows the identified variants, phenotypes, and ocular and systemic complications of patients. The number of affected alleles (monoallelic or biallelic) in each patient was consistent with the inheritance pattern of the affected genes. The phenotypes of the anterior segments related to the *PAX6*, *CYP1B1*, *FOXC1*, and *PITX2* variants are shown in Figure 2. A wide range of phenotypes was observed even with the same affected genes and between the right and left eyes in an individual (Fig 2A-b, C-b, d). Similar phenotypes of aniridia with glaucoma were caused by variants in *PAX6*, *CYPIBI*, and *FOXC1* (Fig 2A-a, B-a, C-a, b).

**Table 2:** Identified Variants and Phenotypes of Patients with ASD/Aniridia

Gene, Inheritance, No. of Patients (Family)	Patient	Sequence Variation cDNA, Protein	Phenotype	Ocular Major Complications	Systemic Abnormalities
*PAX6,* AD 23 (19)	A-1	c.375_376del, p.Arg125SerfsTer7	B-A	L-Gla	(–)
	A-2, 3	c.375_376del, p.Arg125SerfsTer7	B-A	B-Gla	(–)
	(familial)		B-A	(–)	(–)
	A-4	c.140A>G, p.Gln47Arg	B-A	B-Gla, Cat	(–)
	A-5	c.109del, p.Ala37ProfsTer17	B-A	(–)	(–)
	A-6	c.607C>T, p.Arg203Ter	B-A	(–)	(–)
	A-7	c.607C>T, p.Arg203Ter	B-A	B-Gla, Cat	(–)
	A-8, 9	c.765G>C, p.Gln255His	B-A	B-Gla, Cat	(–)
	(familial)		B-A	R-CP, L-Cat	(–)
	A-10	c.77G>A, p.Arg26Gln	R-P	R-M, L-Cat	(–)
	A-11, 12	c.-52+1G>A	B-A	(–)	(–)
	(familial)		B-A	(–)	(–)
	A-13	c.152G>A, p.Gly51Glu	R-AA, L-P	R-Cat	(–)
	A-14	c.949C>T, p.Arg317Ter	B-A	B-Cat	(+)
	A-15	c.949C>T, p.Arg317Ter	B-A	(–)	(–)
	A-16	c.538C>T, p.Gln180Ter	B-A	(–)	(–)
	A-17	c.141G>C, p.Gln47His	B-A	(–)	(–)
	A-18	c.853_854del, p.Ile285SerfsTer5	B-A	(–)	(–)
	A-19	c.54del, p.Arg19GlyfsTer12	B-A	(–)	(–)
	A-20	c.631C > T, p.Gln211Ter	B-A	B-Gla, Cat	(–)
	A-21, 22	c.357+1G>C	B-PA	B-Gla	(–)
	(familial)		B-PA	B-Gla	(–)
	A-23	c.1268A>T, p.Ter423LeuextTer14	B-PA	B-Cat	(–)
*CYP1B1,* AR 11 (11)	A-24	c.970_971dup, p.Thr325SerfsTer104 & c.989_991delCCAinsTCC, p.Ala330_Ser331delinsValArg	B-P	L-PA, B-Gla	(–)
	A-25	c.970_971dup, p.Thr325SerfsTer104 & c.989_991delCCAinsTCC, p.Ala330_Ser331delinsValArg	L-PA	B-Gla	(–)
	A-26	c.1090G>A, p.Val364Met, homo	B-PA	B-P, Gla	(–)
	A-27	c.1269C>G, p.Asn423Lys & c.1090G>A, p.Val364Met	B-P	B-Gla	(–)
	A-28	c.1102C>T, p.Arg368Cys & c.1090G>A, p.Val364Met	B-A	B-Gla, P	(–)
	A-29	c.970_971dup, p.Thr325SerfsTer104, homo	B-P	B-PA, Gla	(–)
	A-30	c.970_971dup, p.Thr325SerfsTer104, homo	B-P	B-A, Gla	(–)
	A-31	c.575A>T, p.Asp192Val & c.512T>C, p.Leu171Pro	R-A, L-PA	B-P, Gla, Cat	(+)
	A-32	c.1331G>A, p.Arg444Gln & c.512T>C, p.Leu171Pro	B-P	B-A, Gla	(–)
	A-33	c.1331G>A, p.Arg444Gln & c.1090G>A, p.Val364Met	B-AA	B-PA, Gla	(–)
	A-34	c.1090G>A, p.Val364Met & c.575A>T, p.Asp192Val	B-P	B-Gla, Cat	(–)
*FOXC1,* AD 7 (7)	A-35	c.367C>T, p.Gln123Ter	R-P, L-AS	R-A	(+)
	A-36	c.140_141dup, p.Ser48ThrfsTer31	B-AA	B-Gla	(+)
	A-37	c.673del, p.Asp225ThrfsTer90	B-AA	B-Gla	(+)
	A-38	c.358C>T, p.Gln120Ter	B-A	B-Gla	(–)
	A-39	c.30del, p.Asn11ThrfsTer34	R-P, L-PA	B-Gla, R-PA, L-AA	(–)
	A-40	c.379C>T, p.Arg127Cys	B-PA	B-AA, Gla	(–)
	A-41	c.484T>C, p.Phe162Leu	B-AA	B-Gla	(–)
*PITX2,* AD 6 (5)	A-42, 43	c.271C>T, p.Arg91Trp	B-AA	(–)	(+)
	(familial)		B-AA	(–)	(+)
	A-44	c.175C>T, p.Gln59Ter	B-AA	R-Gla	(–)
	A-45	c.282del, p.Trp94Ter	B-AA	(–)	(–)
	A-46	c.232C>T, p.Leu78Phe	B-AA	(–)	(+)
	A-47	c.138dup, p.Thr47TyrfsTer152	B-AA	B-BK	(+)
*PITX3,* AD 2 (1)	A-48, 49	c.640_656del	B-P	(–)	(–)
	(familial)	p.Ala214ArgfsTer42	B-P	(–)	(–)
*GJA8,* AD 1 (1)	A-50	c.116C>G, p.Thr39Arg	B-AA	B-Gla, Cat, M	(–)

A = aniridia; AA = Axenfeld anomaly; AD = autosomal dominant; AS = anterior staphyloma; ASD = anterior-segment dysgenesis; AR = autosomal recessive; B = bilateral; BK = bullous keratopathy; Cat = cataract; CP = corneal perforation; Gla = glaucoma; homo = homozygote; L = left eye; M = microphthalmos; P = Peters anomaly; PA = partial aniridia; R = right eye.

Clinical analysis of the variant-positive patients showed that glaucoma, the most severe ocular complication, was present in 15 of 46 eyes (32.6%) with *PAX6* variants, all 22 eyes (100%) with *CYP1B1* variants, 12 of 14 eyes (85.7%) with *FOXC1* variants, 1 of 12 eyes (8.3%) with *PITX2* variants, none of 2 eyes (0%) with *PITX3* variants, and all 2 eyes (100%) with *GJA8* variants. Systemic abnormalities, that is, cardiac disease or hearing loss, were observed in 3 of 7 (42.9%) patients with *FOXC1* variants, and dental abnormalities were noted in 4 of 6 (67%) patients with *PITX2* variants. Autism was observed in 1 of 23 (4.3%) patients with *PAX6* variants and 1 of 11 (9.1%) with *CYP1B1* variants. Two patients with *PITX3* variants and 1 with *GJA8* variants lacked systemic abnormalities.

### Microphthalmia/Anophthalmia

The clinical severity of 28 eyes of 14 index patients with microphthalmia/anophthalmia is shown in Figure 1B. In 7 of 14 patients (50.0%), the severity differed between the right and left eyes. Ocular complications and/or associated ocular anomalies and systemic abnormalities are listed in Supplemental Table 4 (available at www.ophthalmologyscience.org). Among the 14 patients, 2 (14.3%) had a family history, but the phenotypes differed: one case involved a mother with unilateral microphthalmos, optic nerve aplasia, and coloboma and the other involved a father with congenital cataracts and microphthalmia.

Pathogenic variants in 5 genes were identified in 5 of 14 families (35.7%). Table 3 shows the causative genes identified for clinical diagnoses. The genetic diagnosis rate was high for anophthalmia and extreme microphthalmia.

**Table 3:** Pathogenic Variants Identified in Patients with Microphthalmia/Anophthalmia (28 Eyes of 14 Patients)

Clinical Diagnosis∗ (No. of Eyes)	Affected Genes (No. of Eyes)	Total No. of Eyes with Pathogenic Variants (Diagnostic Rate)
Microphthalmia (15)	*BMP4* (1) *RARB* (1) *SOX2* (1)	3 (20.0%)
Extreme microphthalmia (8)	*ABCB6* (2) *BMP4* (1) *RARB* (1)	4 (50.0%)
Anophthalmia (5)	*OTX2* (2) *SOX2* (1)	3 (60.0%)

∗ Seven of 14 patients exhibited a difference in severity between the right and left eyes.

The detected variants for microphthalmia/anophthalmia in this study are shown in Supplemental Table 5 (available at www.ophthalmologyscience.org). All 5 variants, except the *BMP4* variant, were assessed as pathogenic or likely pathogenic according to the American College of Medical Genetics and Genomics guidelines. The *BMP4* variant was assessed as deleterious by all in-silico programs tested. The variants of *RARB* and *SOX2* were de novo. The *ABCB6* and *BMP4* variants were shared by unaffected mothers, and the *OTX2* variant was present in a mother with unilateral microphthalmia, coloboma, persistent fetal vasculature, and optic nerve aplasia. The variants of *OTX2* (c.266delG), *RARB* (c.844G>T), and *SOX2* (c.137A>T) were previously unreported, and the variants of *ABCB6* (c.1992A>T) and *BMP4* (c.722T>A) have been reported only in East Asia.

Table 4 shows the identified variants, phenotypes, and ocular and systemic complications. The severities of microphthalmia/anophthalmia varied among patients, even with the same affected genes, and between the right and left eyes of an individual. Of the 5 patients, 2 patients with *BMP4* and *RARB* variants presented with systemic abnormalities related to neurologic disorders.

**Table 4:** Identified Variants, Phenotypes, and Complications of Patients with Microphthalmia/Anophthalmia

Gene	Patient, (No. of Families)	Mode of Inheritance	Sequence Variation cDNA, Protein	Phenotype	Ocular Complications	Systemic Abnormalities
*ABCB6*	M-1 (1)	AD unaffected maternal, nonpenetrant	c.1992A>T, p.Asp641Val	B-extreme microphthalmia	(–)	(–)
*BMP4*	M-2 (1)	AD unaffected maternal, nonpenetrant	c.722T>A, p.Leu241His	R-extreme microphthalmos, L-microphthalmos	L-iris and chorioretinal coloboma, persistent fetal vasculature	Epilepsy, neurologic disorders
*OTX2*	M-3 (1)	AD maternal	c.266del, p.Arg89GlnfsTer20	B-anophthalmia	(–)	(–)
*RARB*	M-4 (1)	AD de novo	c.844G>T, p.Gly282Cys	R-microphthalmos, L-extreme microphthalmos	R-Peters anomaly type II	Neurologic disorders
*SOX2*	M-5 (1)	AD de novo	c.137A>T, p.Asn46Ile	R-microphthalmos L-anophthalmos	R-Peters anomaly	(–)

AD = autosomal dominant; B = bilateral; L = left eye; R = right eye.

All findings for variant-positive patients and their parents are listed in Supplemental Table 6 (available at www.ophthalmologyscience.org).

## Discussion

We conducted molecular and clinical analysis for a large cohort of patients with bilateral ASD/aniridia and microphthalmia/anophthalmia. A total of 111 patients and their family members were recruited through a multicenter collaborative study in Japan. Using custom-designed NGS panels, we achieved genetic diagnosis rates of 50.0% for bilateral ASD/aniridia and 37.5% for microphthalmia/anophthalmia. The current panel of genes used in this study, while beneficial for clinical genetic diagnosis of these diseases, may be improved in the future by the addition of other genes that contribute to a smaller percentage of cases. In particular, our data highlight the clinical importance of 6 genes (*PAX6*, *CYP1B1*, *FOXC1*, *PITX2*, *PITX3*, and *GJA8*) as causative genes for ASD/aniridia. Previously, Patel et al conducted an “oculome” panel test involving 429 eye disease-associated genes and reported that detection rates were 24.8% for ASD and 8.2% for microphthalmia-anophthalmia-coloboma.^7^ Likewise, a gene panel test by Neuhann et al involving 186 eye-related genes yielded detection rates of 43% for ASD/primary congenital glaucoma and 41% for microphthalmia-anophthalmia-coloboma.^17^ The present study demonstrated the usefulness of NGS panels focusing on the major causative genes in clinical practice for bilateral ASD/aniridia and microphthalmia/anophthalmia.

We found that most variants of autosomal-dominant ASD/aniridia, except for those in *FOXC1*, were de novo. This study showed high penetrance of pathogenic variants in *PAX6*, *PITX2*, and *GJA8* and incomplete penetrance of variants in *FOXC1.* In contrast, microphthalmia/anophthalmia‑associated variants in *ABCB6*, *BMP4*, and *OTX2* were predominantly inherited from parents with no or different ocular phenotypes. Consistent with this, variable penetrance and phenotypic diversity have been reported in several congenital ocular malformations.^18^, ^19^, ^20^, ^21^ These findings improve the accuracy of genetic counseling, including the estimation of recurrence risk.

We identified 11 previously unreported variants in 11 patients. Among these, the *RARB* variant was reported in our recent paper.^8^ Five of them were confirmed as de novo variants in *FOXC1*, *PITX2*, *RARB*, and *SOX2*. These findings emphasize the importance of de novo variants as the genetic causes of autosomal-dominant disorders. Furthermore, we identified several variants that have been identified only in East Asian populations. These variants were most commonly identified in *CYP1B1*, indicating that ethnicity-specific founder mutations are involved in the etiology of autosomal-recessive ASD/aniridia.

The present study provided useful information about phenotypic diversity and the frequency of ocular and systemic complications. Our genetic study confirmed that various phenotypes classified as ASD, such as Peters anomaly and Axenfeld anomaly, constitute a group of disorders on the same spectrum and may overlap with aniridia. We also found that ASD/aniridia exhibits genetic heterogeneity.

Most importantly, the incidence of glaucoma, a severe ocular complication, in patients with ASD/aniridia varied among patients depending on the causative genes. Our results indicate that the risk of early-onset glaucoma is high in patients with variants in *CYP1B1* or *FOXC1* and low in patients with variants in *PITX2* or *PITX3*. Consistent with this, Fuse et al reported that *CYP1B1* and *FOXC1* were the major causative genes of childhood glaucoma in Japan.^22^ We also found that the frequency of systemic complications was relatively high in patients with *FOXC1* or *PITX2* and relatively low in patients with variants in *PAX6*, *CYP1B1*, *PITX3*, or *GAJ8.* These results support the previously proposed notion^23^ that patients with eye diseases due to *FOXC1* or *PITX2* variants should be managed in collaboration with pediatricians. However, among the 5 variant-positive patients with microphthalmia/anophthalmia, only 2 had systemic complications and they carried *BMP4* and *RARB* variants. The current results imply that genetic diagnoses of ASD/aniridia and microphthalmia/anophthalmia are useful to manage the ocular and systemic complications. Furthermore, genetic diagnosis would increase the understanding of the underlying mechanism of these disorders and facilitate parents’ proper acceptance of their child’s disease.

### Limitations

This study had several limitations. First, our NGS panels included only a small number of genes. The usefulness of these panels will increase by adding other major causative genes. Second, in some cases, parental blood samples were unavailable. Therefore, the exact penetrance for each gene abnormality could not be determined. Finally, the incidence of ocular and systemic complications may be underestimated, because the study population consists primarily of children. To facilitate further clinical applications, long-term observation of the natural course of the disease and ocular and systemic complications is necessary for each causative gene.

## Conclusions

The results of the present study highlighted the usefulness of NGS panel analyses for the clinical diagnosis of ASD/aniridia and microphthalmia/anophthalmia. Indeed, despite targeting only a small number of genes, our NGS panels achieved diagnostic rates of 50.0% for ASD/aniridia and 37.5% for microphthalmia/anophthalmia. Moreover, this study significantly broadened the mutation spectrum and phenotypic variation of these diseases. Our data indicate that de novo variants in *PAX6*, *FOXC1*, *PITX2*, and *GJA8* account for a certain percentage of the etiology of autosomal-dominant ASD/aniridia. This study provides the first evidence that *CYP1B1* founder mutations contribute to the development of autosomal-recessive ASD/aniridia in Japan. Furthermore, variants in *ABCB6*, *BMP4*, and *OTX2* inherited from parents with no or different ocular phenotypes are likely to play major roles in the development of microphthalmia/anophthalmia. More importantly, we found a high incidence of glaucoma in patients with *CYP1B1* and *FOXC1* variants and frequent systemic abnormalities in patients with *FOXC1* and *PITX2* variants. In addition, *BMP4* and *RARB* variants appear to cause neurologic abnormalities. The current results contribute to better management and care for children with these rare intractable eye diseases.
