# Supplementary material for: Molecular and Clinical Analyses of 111 Patients with Bilateral Anterior-Segment Dysgenesis/Aniridia and Microphthalmia/Anophthalmia
Source: Ophthalmol Sci. 2026 Jul 28;6(10):101339. doi: 10.1016/j.xops.2026.101339 (PMC13524468; doi:10.1016/j.xops.2026.101339)
Supplement: Supplementary file 1 [file mmc1.pdf]

Supplemental Table 1. Target Genes of Panels for ASD/Aniridia and Microphthalmia/Anophthalmia

**Genes for ASD/Aniridia**	**Mode of Inheritance**	**Genes for Microphthalmia**	**Mode of Inheritance**
B3GLCT	AR	*ABCB6*	AD
COL4A1	AD	*ALDH1A3*	AR
CPAMD8	AR	*BMP4*	AD
CYP1B1	AR	*CRYBA4*	AD
FOXC1	AD	*GDF3*	AD
FOXE3	AR, (AD)	*OTX2*	AD
GJA8	AD	*RARB*	AD, (AR)
PAX6	AD	*RAX*	AR
PITX2	AD	*SOX2*	AD
PITX3	AD, (AR)	*STRA6*	AR
PXDN	AR	*TENM3*	AR
		VSX2	AR

AD = autosomal dominant; AR = autosomal recessive.
