# Supplementary material for: Molecular and Clinical Analyses of 111 Patients with Bilateral Anterior-Segment Dysgenesis/Aniridia and Microphthalmia/Anophthalmia
Source: Ophthalmol Sci. 2026 Jul 28;6(10):101339. doi: 10.1016/j.xops.2026.101339 (PMC13524468; doi:10.1016/j.xops.2026.101339)
Supplement: Supplementary file 2 [file mmc2.pdf]

Supplemental Table 2. Ocular Complications, Associated Ocular Anomalies, and Systemic Abnormalities with ASD/Aniridia (194 Eyes of 97 Patients).

**Clinical Diagnosis of ASD/Aniridia N=194 eyes**	**Ocular Complication/ Associated Ocular Anomalies**	**No. eyes**	**No. Eyes with Complications**
Peters anomaly N=70 eyes	Glaucoma	32 (45.7%)	48 (68.6%)
	Iris hypoplasia	13 (18.6%)	
	Microcornea	9 (12.9%)	
	Cataracts	7 (10.0%)	
	Vitreoretinal diseases	5 (7.1%)	
	Corneal perforation	1 (1.4%)	
Axenfeld anomaly N=39 eyes	Glaucoma	21 (52.8%)	24 (61.5%)
	Microcornea	8 (20.5%)	
	Cataracts	5 (12.8%)	
	Iris hypoplasia	4 (10.3%)	
	Bullous keratopathy	3 (7.7%)	
	Vitreoretinal diseases	2 (5.1%)	
	Lens dislocation	1 (2.6%)	
Sclerocornea N=10 eyes	Glaucoma	2 (20.0%)	2 (20.0%)
Aniridia N=54 eyes	Glaucoma	27 (50.0%)	34 (63.0%)
	Cataracts	20 (37.0%)	
	Corneal opacity	8 (14.8%)	
	Lens dislocation	2 (3.7%)	
	Retinal detachment	2 (3.7%)	
	Corneal perforation	1 (1.9%)	
Partial aniridia N=14 eyes	Glaucoma	11 (78.6%)	13 (92.9%)
	Cataracts	3 (21.4%)	
	Central corneal opacity	3 (21.4%)	
	Posterior embryotoxon	3 (21.4%)	

**ASD/Aniridia N=97 Patients**	**Systemic Abnormalities**	**No. patients**	**Total Number**
	Heart disease	11 (11.3%)	29 (29.9%)
	Dental anomalies	7 (7.2%)	
	Neurological disorders	5 (5.2%)	
	Developmental delay	4 (4.1%)	
	Kidney disease	3 (3.1%)	
	Hearing impairment	3 (3.1%)	
	Cleft lip and palate	3 (3.1%)	

ASD = anterior-segment dysgenesis.
