# Supplementary material for: Molecular and Clinical Analyses of 111 Patients with Bilateral Anterior-Segment Dysgenesis/Aniridia and Microphthalmia/Anophthalmia
Source: Ophthalmol Sci. 2026 Jul 28;6(10):101339. doi: 10.1016/j.xops.2026.101339 (PMC13524468; doi:10.1016/j.xops.2026.101339)
Supplement: Supplementary file 3 [file mmc3.pdf]

Supplemental Table 3. Detected Variants for ASD/Aniridia

Gene	Variant cDNA	Protein	ACMG	Number of Patients (Family)	De Novo /Familial	Previously Reported* /Novel
PAX6	c.375_376del	p.Arg125SerfsTer7	P	3 (2)	De novo/ Paternal	Previously reported
	c.140A>G	p.Gln47Arg	P	1 (1)	De novo	Previously reported
	c.109del	p.Ala37ProfsTer17	P	1 (1)	De novo	Previously reported
	c.607C>T	p.Arg203Ter	P	2 (2)	Maternal /nc	Previously reported
	c.765G>C	p.Gln255His	P	2 (1)	Paternal suspected	Previously reported
	c.77G>A	p.Arg26Gln	LP	1 (1)	De novo	Previously reported
	c.-52+1G>A		LP	2 (1)	Maternal	Previously reported
	c.152G>A	p.Gly51Glu	LP	1 (1)	nc	Previously reported
	c.949C>T	p.Arg317Ter	P	2 (2)	Paternal /nc	Previously reported
	c.538C>T	p.Gln180Ter	P	1 (1)	De novo	Previously reported
	c.141G>C	p.Gln47His	P	1 (1)	nc	Novel
	c.853_854del	p.Ile285SerfsTer5	P	1 (1)	nc	Novel
	c.54del	p.Arg19GlyfsTer12	P	1 (1)	nc	Previously reported
	c.631C>T	p.Gln211Ter	LP	1 (1)	nc	Previously reported
	c.357+1G>C		P	2 (1)	Maternal	Previously reported
	c.1268A>T	p.Ter423LeuextTer14	P	1 (1)	De novo	Previously reported
CYP1B1	c.970_971dup	p.Thr325SerfsTer104	P	6 (4)	Maternal 3 Paternal 3	Previously reported
	c.989_991delCCAinsTCC	p.Ala330_Ser331delinsValArg	LP	2 (2)	nc 1 Maternal 1	Previously reported
	c.1090G>A	p.Val364Met	LP	6 (5)	Maternal 2 Paternal 4	Previously reported
	c.1269C>G	p.Asn423Lys	VUS†	1 (1)	Maternal	Previously reported

	c.1102C>T	p.Arg368Cys	P	1 (1)	Paternal	Previously reported
	c.575A>T	p.Asp192Val	LP	2 (2)	Maternal 2	Previously reported
	c.512T>C	p.Leu171Pro	VUS†	2 (2)	Paternal 2	Previously reported
	c.1331G>A	p.Arg444Gln	P	2 (2)	Maternal 2	Previously reported
FOXC1	c.367C>T	p.Gln123Ter	P	1 (1)	Unaffected maternal	Previously reported
	c.140_141dup	p.Ser48ThrfsTer31	LP	1 (1)	De novo	Novel
	c.673del	p.Asp225ThrfsTer90	LP	1 (1)	De novo	Novel
	c.358C>T	p.Gln120Ter	P	1 (1)	De novo	Previously reported
	c.30del	p.Asn11ThrfsTer34	LP	1 (1)	De novo	Previously reported
	c.379C>T	p.Arg127Cys	LP	1 (1)	De novo	Previously reported
	c.484T>C	p.Phe162Leu	LP	1 (1)	Unaffected maternal	Novel
PITX2	c.271C>T	p.Arg91Trp	P	2 (1)	Paternal suspected	Previously reported
	c.175C>T	p.Gln59Ter	LP	1 (1)	Maternal	Previously reported
	c.282del	p.Trp94Ter	LP	1 (1)	De novo	Novel
	c.232C>T	p.Leu78Phe	LP	1 (1)	nc	Novel
	c.138dup	p.Thr47TyrfsTer152	LP	1 (1)	nc	Novel
PITX3	c.640_656del	p.Ala214ArgfsTer42	P	2 (1)	Paternal	Previously reported
GJA8	c.116C>G	p.Thr39Arg	LP	1 (1)	De novo	Previously reported

ASD = anterior-segment dysgenesis; P = pathogenic; LP = likely pathogenic; VUS = variant of uncertain significance; nc = not checked.

*Evaluated using HGMD, ClinVar, gnomAD, and UCSC.

†Predicted to cause protein functional changes in in-silico analysis using Mutation Taster, SIFT, and PolyPhen2.
