# Supplementary material for: Molecular and Clinical Analyses of 111 Patients with Bilateral Anterior-Segment Dysgenesis/Aniridia and Microphthalmia/Anophthalmia
Source: Ophthalmol Sci. 2026 Jul 28;6(10):101339. doi: 10.1016/j.xops.2026.101339 (PMC13524468; doi:10.1016/j.xops.2026.101339)
Supplement: Supplementary file 4 [file mmc4.pdf]

Supplemental Table 4. Ocular Complications, Associated Ocular Anomalies, and Systemic Abnormalities with Microphthalmia/Anophthalmia (28 Eyes of 14 Patients).

**Clinical Diagnosis of Microphthalmia N=28 eyes**	**Ocular Complication/ Associated Ocular Anomalies No. eyes**		**Total No. Eyes with Complications**
Microphthalmia N=15 eyes	Coloboma	13 (86.7%)	15 (100.0%)
	Persistent fetal vasculature	5 (33.3%)	
	ASD	5 (33.3%)	
	Lens anomaly	4 (26.7%)	
	Corneal opacity	2 (13.3%)	
	Disc anomaly	2 (13.3%)	
Extreme microphthalmia N=8 eyes	Difficult to evaluate		
Anophthalmia N=5 eyes	Difficult to evaluate		

**Microphthalmia N=14 patients**	**Systemic Abnormalities No. patients**		**Total No. Patients with Complications**
	Multiple anomalies	2 (14.3%)	5 (35.7%)
	Epilepsy and neurologic disorders	2 (14.3%)	
	Hearing impairment	1 (7.1%)	

ASD = anterior-segment dysgenesis.
