# Supplementary material for: Molecular and Clinical Analyses of 111 Patients with Bilateral Anterior-Segment Dysgenesis/Aniridia and Microphthalmia/Anophthalmia
Source: Ophthalmol Sci. 2026 Jul 28;6(10):101339. doi: 10.1016/j.xops.2026.101339 (PMC13524468; doi:10.1016/j.xops.2026.101339)
Supplement: Supplementary file 5 [file mmc5.pdf]

Supplemental Table 5. Detected Variants for Microphthalmia/Anophthalmia

Gene	Variant cDNA	Protein	ACMG	No. Patients (Families)	De Novo /Familial	Previously Reported*/Novel
ABCB6	c.1992A>T	p.Asp641Val	LP	1 (1)	Unaffected maternal	Previously reported
BMP4	c.722T>A	p.Leu241His	VUS†	1 (1)	Unaffected maternal	Previously reported
OTX2	c.266delG	p.Arg89GlnfsTer20	LP	1 (1)	Maternal	Novel
RARB	c.844G>T	p.Gly282Cys	LP	1 (1)	De novo	Novel‡
SOX2	c.137A>T	p.Asn46Ile	LP	1 (1)	De novo	Novel

LP = likely pathogenic; VUS = variant of uncertain significance; ACMG = American College of Medical Genetics and Genomics.
*Evaluated using HGMD, ClinVar, gnomAD, and UCSC.
†Predicted to cause protein functional changes in in-silico analysis using Mutation Taster, SIFT, and PolyPhen2.
‡This variant has been reported in our recent paper.[8]
