# Supplementary material for: Molecular and Clinical Analyses of 111 Patients with Bilateral Anterior-Segment Dysgenesis/Aniridia and Microphthalmia/Anophthalmia
Source: Ophthalmol Sci. 2026 Jul 28;6(10):101339. doi: 10.1016/j.xops.2026.101339 (PMC13524468; doi:10.1016/j.xops.2026.101339)
Supplement: Supplementary file 6 [file mmc6.pdf]

Supplemental Table 6. List of All Findings for Variant-Positive Patients and Their Parents

Gene, Inheritance, No. Patients (Family)	Patient	Sequence Variation cDNA, Protein	Phenotype	Ocular Major Complications	Systemic Abnormalities, Specifics	Parents' Genetic Test Results	Phenotype of Affected Parent
PAX6,	A-1	c.375_376del, p.Arg125SerfsTer7	B-A	L-Gla	(-)	Fa: (-)	
AD						Mo: (-)	
23 (19)	A-2, 3	c.375_376del, p.Arg125SerfsTer7	B-A	B-Gla	(-)	Fa: nc	
	(familial, Mo)		B-A	(-)	(-)	Mo: c.375_376del	B-A
	A-4	c.140A>G, p.Gln47Arg	B-A	B-Gla, Cat	(-)	Fa: (-)	
						Mo: (-)	
	A-5	c.109del, p.Ala37ProfsTer17	B-A	(-)	(-)	Fa: (-)	
						Mo: (-)	
	A-6	c.607C>T, p.Arg203Ter	B-A	(-)	(-)	Fa: nc	
						Mo: c.607C>T	B-A, Cat
	A-7	c.607C>T, p.Arg203Ter	B-A	B-Gla, Cat	(-)	Fa: nc	
						Mo: nc	
	A-8, 9	c.765G>C, p.Gln255His	B-A	B-Gla, Cat	(-)	Fa: nc	
	(familial)		B-A	R-CP, L-Cat	(-)	Mo: nc	
	A-10	c.77G>A, p.Arg26Gln	R-P	R-M, L-Cat	(-)	Fa: (-)	
						Mo: (-)	
	A-11, 12	c.-52+1G>A	B-A	(-)	(-)	Fa: nc	
	(familial)		B-A	(-)	(-)	Mo: nc	B-A, Cat, Gla
	A-13	c.152G>A, p.Gly51Glu	R-AA, L-P	R-Cat	(-)	Fa: nc	
						Mo: nc	
	A-14	c.949C>T, p.Arg317Ter	B-A	B-Cat	(+) Autism	Fa: nc	
						Mo: nc	
	A-15	c.949C>T, p.Arg317Ter	B-A	(-)	(-)	Fa: c.949C>T	B-A
						Mo: (-)	
	A-16	c.538C>T, p.Gln180Ter	B-A	(-)	(-)	Fa: (-)	
						Mo: (-)	
	A-17	c.141G>C, p.Gln47His	B-A	(-)	(-)	Fa: nc	
						Mo: nc	
	A-18	c.853_854del, p.Ile285SerfsTer5	B-A	(-)	(-)	Fa: nc	
						Mo: nc	
	A-19	c.54del, p.Arg19GlyfsTer12	B-A	(-)	(-)	Fa: nc	
						Mo: nc	
	A-20	c.631C>T, p.Gln211Ter	B-A	B-Gla, Cat	(-)	Fa: nc	
						Mo: nc	

	A-21, 22<br>(familial, Mo)	c.357+1G>C	B-PA<br>B-PA	B-Gla<br>B-Gla	(-)<br>(-)	Fa: nc<br>Mo: c.357+1G>C	B-PA, Gla
	A-23	c.1268A>T, p.Ter423LeuextTer14	B-PA	B-Cat	(-)	Fa: (-)<br>Mo: (-)	
CYP1B1,<br>AR<br>11 (11)	A-24	c.970_971dup,<br>p.Thr325SerfsTer104<br>& c.989_991delCCAinsTCC,<br>p.Ala330_Ser331delinsValArg	B-P	L-PA, B-Gla	(-)	Fa: nc<br>Mo: c.970_971dup	
	A-25	c.970_971dup,<br>p.Thr325SerfsTer104<br>& c.989_991delCCAinsTCC,<br>p.Ala330_Ser331delinsValArg	L-PA	B-Gla	(-)	Fa: c.970_971dup<br>Mo:<br>c.989_991delCCA<br>insTCC	
	A-26	c.1090G>A, p.Val364Met, homo	B-PA	B-P, Gla	(-)	Fa: c.1090G>A<br>Mo: c.1090G>A	
	A-27	c.1269C>G, p.Asn423Lys<br>& c.1090G>A, p.Val364Met	B-P	B-Gla	(-)	Fa: c.1090G>A<br>Mo: c.1269C>G	
	A-28	c.1102C>T, p.Arg368Cys<br>& c.1090G>A, p.Val364Met	B-A	B-Gla, P	(-)	Fa: c.1102C>T<br>Mo: c.1090G>A	
	A-29	c.970_971dup,<br>p.Thr325SerfsTer104, homo	B-P	B-PA, Gla	(-)	Fa: c.970_971dup<br>Mo: c.970_971dup	
	A-30	c.970_971dup,<br>p.Thr325SerfsTer104, homo	B-P	B-A, Gla	(-)	Fa: c.970_971dup<br>Mo: c.970_971dup	
	A-31	c.575A>T, p.Asp192Val<br>& c.512T>C, p.Leu171Pro	R-A, L-PA	B-P, Gla, Cat	(+) Autism	Fa: c.512T>C<br>Mo: c.575A>T	
	A-32	c.1331G>A, p.Arg444Gln<br>& c.512T>C, p.Leu171Pro	B-P	B-A, Gla	(-)	Fa: c.512T>C<br>Mo: c.1331G>A	
	A-33	c.1331G>A, p.Arg444Gln<br>& c.1090G>A, p.Val364Met	B-AA	B-PA, Gla	(-)	Fa: c.1090G>A<br>Mo: c.1331G>A	
	A-34	c.1090G>A, p.Val364Met<br>& c.575A>T, p.Asp192Val	B-P	B-Gla, Cat	(-)	Fa: c.1090G>A<br>Mo: c.575A>T	
FOXC1,<br>AD<br>7 (7)	A-35	c.367C>T, p.Gln123Ter	R-P, L-AS	R-A	(+) Cardiac disease	Fa: (-)<br>Mo: c.367C>T	Phenotype (-)
	A-36	c.140_141dup, p.Ser48ThrfsTer31	B-AA	B-Gla	(+) Cardiac disease<br>Kidney disease	Fa: (-)<br>Mo: (-)	
	A-37	c.673del, p.Asp225ThrfsTer90	B-AA	B-Gla	(+) Cardiac disease<br>Hearing loss	Fa: (-)<br>Mo: (-)	
	A-38	c.358C>T, p.Gln120Ter	B-A	B-Gla	(-)	Fa: (-)<br>Mo: (-)	
	A-39	c.30del, p.Asn11ThrfsTer34	R-P, L-PA	B-Gla,<br>R-PA, L-AA	(-)	Fa: (-)<br>Mo: (-)	

	A-40	c.379C>T, p.Arg127Cys	B-PA	B-AA, Gla	(-)	Fa: (-)<br>Mo: (-)	
	A-41	c.484T>C, p.Phe162Leu	B-AA	B-Gla	(-)	Fa: (-)<br>Mo: c.484T>C	Phenotype (-)
PITX2,<br>AD<br>6 (5)	A-42, 43<br>(familial)	c.271C>T, p.Arg91Trp	B-AA<br>B-AA	(-)<br>(-)	(+) Dental anomalies<br>(+) Dental anomalies	Fa: nc<br>Mo: (-)	
	A-44	c.175C>T, p.Gln59Ter	B-AA	R-Gla	(-)	Fa: (-)<br>Mo: c.175C>T	B-PA, Gla
	A-45	c.282del, p.Trp94Ter	B-AA	(-)	(-)	Fa: (-)<br>Mo: (-)	
	A-46	c.232C>T, p.Leu78Phe	B-AA	(-)	(+) Dental anomalies	Fa: nc<br>Mo: nc	
	A-47	c.138dup, p.Thr47TyrfsTer152	B-AA	B-BK	(+) Dental anomalies	Fa: nc<br>Mo: nc	
PITX3,<br>AD<br>2 (1)	A-48, 49<br>(familial)	c.640_656del, p.Ala214ArgfsTer42	B-P<br>B-P	(-)<br>(-)	(-)<br>(-)	Fa: c.640_656del<br>Mo: (-)	Congenital Cat
GJA8,<br>AD<br>1 (1)	A-50	c.116C>G, p.Thr39Arg	B-AA	B-Gla, Cat, M	(-)	Fa: (-)<br>Mo: (-)	
ABCB6,<br>AD<br>1 (1)	M-1	c.1992A>T, p.Asp641Val	B-EM	(-)	(-)	Fa: (-)<br>Mo: c.1992A>T	Phenotype (-)
BMP4,<br>AD<br>1 (1)	M-2	c.722T>A, p.Leu241His	R-EM,<br>L-M	L-CO, PFV	(+) Neurologic disorders	Fa: nc<br>Mo: c.722T>A	Phenotype (-)
OTX2,<br>AD<br>1 (1)	M-3	c.266delG, p.Arg89GlnfsTer20	B-AN	(-)	(-)	Fa: (-)<br>Mo: c.266delG	R-M, ONA, CO
RARB,<br>AD<br>1 (1)	M-4	c.844G>T, p.Gly282Cys	R-M,<br>L-EM	R-P	(+) Neurologic disorders	Fa: (-)<br>Mo: (-)	
SOX2,<br>AD<br>1 (1)	M-5	c.137A>T, p.Asn46Ile	R-M,<br>L-AN	R-P	(-)	Fa: (-)<br>Mo: (-)	

ASD = anterior-segment dysgenesis; AD = autosomal dominant; AR = autosomal recessive; homo = homozygote; R = right eye; L = left eye; B = bilateral; P = Peters anomaly; AA = Axenfeld anomaly; A = aniridia; PA = partial aniridia; AS = anterior staphyloma; Gla = glaucoma; Cat = cataract; CP = corneal perforation; BK = bullous keratopathy; M = microphthalmos; EM = extreme microphthalmos; AN = anophthalmos; CO = coloboma; PFV = persistent fetal vasculature; ONA = optic nerve aplasia; Fa = father, Mo = mother, nc = not checked.
